# Correspondence and Clinical Reports

**Published:** 1881-10

**Authors:** 


### Correspondence and Clinical Reports.

We publish in this number a letter addressed to the editors by a member of the profession of this city. It will be observed that reference is made to the reply of our esteemed editorial predecessor, in which he promised, "with penitence and prayer," to revoke his steps in admitting a certain communication for publication. The present editors desire to say that they, so far from wishing to exclude, on the contrary, are anxious to receive, communications upon all subjects of interest to the profession. Especially do they desire once more to call the attention of their medical brethren to the importance of clinical reports, and to urge them to renewed zeal in compiling and transmitting them for publication. It is to the profession of this city and the neighboring country that the Journal must look for active support, and we earnestly hope that by this means the number and character of the clinical reports may be made a principal and interesting feature. In conclusion we have only one word of caution to add, that all communications be as brief and concise as is consistent with perspicuity and completeness.

